# SWI/SNF复合体基因突变促进NSCLC细胞在NSI小鼠体内肝转移的研究

**DOI:** 10.3779/j.issn.1009-3419.2023.102.35

**Published:** 2023-10-20

**Authors:** Lingling GAO, Zhi XIE, Shouheng LIN, Zhiyi LV, Wenbin ZHOU, Ji CHEN, Linlin ZHU, Li ZHANG, Penghui ZENG, Xiaodan HUANG, Wenqing YAN, Yu CHEN, Danxia LU, Shuilian ZHANG, Weibang GUO, Peng LI, Xuchao ZHANG

**Affiliations:** ^1^510080 广州，南方医科大学附属广东省人民医院（广东省医学科学院）; ^1^Guangdong Provincial People's Hospital (Guangdong Academy of Medical Sciences), Southern Medical University, Guangzhou 510080, China; ^2^510515 广州，南方医科大学第二临床医学院; ^2^The Second School of Clinical Medicine, Southern Medical University, Guangzhou 510515, China; ^3^510080 广州，南方医科大学附属广东省人民医院（广东省医学科学院），医学研究部，广东省肺癌转化医学重点实验室; ^3^Guangdong Provincial Key Laboratory of Translational Medicine in Lung Cancer, Medical Research Center, Guangdong Provincial People's Hospital (Guangdong Academy of Medical Sciences), Southern Medical University, Guangzhou 510080, China; ^4^510530 广州，中国科学院广州生物医药与健康研究院; ^4^Guangzhou Institutes of Biomedicine and Health, Chinese Academy of Sciences, Guangzhou 510530, China; ^5^510006 广州，华南理工大学医学院; ^5^School of Medicine, South China University of Technology, Guangzhou 510006, China

**Keywords:** 肺肿瘤, SWI/SNF复合体, 突变, 肿瘤转移, Lung neoplasms, SWI/SNF complex, Mutation, Neoplasm metastasis

## Abstract

**背景与目的:**

SWI/SNF复合体（switch/sucrose nonfermentable chromatin-remodeling complex, SWI/SNF）是一种重要的染色质重塑复合物，其亚基变异在多种肿瘤中存在，并与多种肿瘤细胞生物学特征相关。但其基因突变是否参与非小细胞肺癌（non-small cell lung cancer, NSCLC）肝转移过程尚不清楚。本研究拟探究SWI/SNF复合体基因突变对NSCLC肝转移的影响及潜在机制。

**方法:**

我们使用全外显子组测序（whole-exome sequencing, WES）分析了NSCLC细胞H1299、H23和H460中SWI/SNF复合体基因突变。通过CRISPR/Cas9（clustered regularly interspaced short palindromic repeats）技术构建了ARID1A基因稳定敲除的H1299细胞株，建立了小鼠模型模拟NSCLC肝转移，观察不同基因突变对肝转移的影响。利用RNA-Seq和蛋白印迹分析差异基因的表达，并通过免疫组化技术（immunohistochemistry, IHC）检测了SWI/SNF复合体调控的靶分子在小鼠肝转移灶中的表达。

**结果:**

WES分析确定了SWI/SNF复合体基因的突变情况。动物实验结果显示SWI/SNF复合体基因突变与免疫缺陷小鼠较高的肝转移率相关。转录组测序和蛋白印迹分析显示SWI/SNF复合体基因突变细胞中ALDH1A1和APOBEC3B表达上调，尤其是ARID1A蛋白缺失的H460和H1299 sgARID1A中ALDH1A1表达水平显著上升。IHC染色亦显示H460和H1299 sgARID1A细胞肝转移灶中ALDH1A1高表达。

**结论:**

本研究强调了SWI/SNF复合体基因ARID1A和SMARCA4等突变在促进肺癌细胞肝转移中的关键作用。这些基因突变可能通过促进ALDH1A1与APOBEC3B高表达进而发挥肝特异性转移的作用，为深入探究肺癌肝转移分子机制提供了新线索。

## Abstract (en)

**Background and objective:**

The switch/sucrose nonfermentable chromatin-remodeling (SWI/SNF) complex is a pivotal chromatin remodeling complex, and the genomic alterations (GAs) of the SWI/SNF complex are observed in several cancer types, correlating with multiple biological features of tumor cells. However, their role in liver metastasis of non-small cell lung cancer (NSCLC) remains unclear. Our study aims to investigate the role and potential mechanisms underlying NSCLC liver metastasis induced by the GAs of SWI/SNF complex.

**Methods:**

The GAs of SWI/SNF complex in NSCLC cell lines (H1299, H23 and H460) were identified by whole-exome sequencing (WES). ARID1A knockout H1299 cell was constructed with the CRISPR/Cas9 technology. The mouse model of liver metastasis from NSCLC was established to simulate lung cancer liver metastasis and observe the metastasis rate under different gene mutation conditions. RNA sequencing and Western blot were conducted for differential gene expression analysis. Immunohistochemistry (IHC) analysis was used to assess protein expression levels of SWI/SNF-regulated target molecules in mouse liver metastases.

**Results:**

WES analysis revealed intracellular gene mutations. The animal experiments demonstrated a correlation between the GAs of SWI/SNF complex and a higher liver metastasis rate in immunodeficient mice. Transcriptome sequencing and Western blot analysis showed upregulated expression of ALDH1A1 and APOBEC3B in SWI/SNF-mut cells, particularly in ARID1A-deficient H460 and H1299 sgARID1A cells. IHC staining of mouse liver metastases further demonstrated elevated expression of ALDH1A1 in the H460 and H1299 sgARID1A group.

**Conclusion:**

This study underscores the critical role of the GAs of SWI/SNF complex, such as ARID1A and SMARCA4, in promoting liver metastasis of lung cancer cells. The GAs of SWI/SNF complex may promote liver-specific metastasis by upregulating ALDH1A1 and APOBEC3B expression, providing novel insights into the molecular mechanisms underlying lung cancer liver metastasis.

肺癌是高发病率和高死亡率的主要癌种。非小细胞肺癌（non-small cell lung cancer, NSCLC）在肺癌病例中占比约为85%^[1]^。肺癌的复发转移是导致患者死亡的主要因素之一，而在NSCLC患者中，肝脏是最常见的远处转移部位之一，据报道晚期NSCLC患者中肝转移发生率约为20%^[2,3]^，与其他器官转移相比，晚期肝转移患者死亡率最高^[4]^，且预后较差，中位生存期（overall survival, OS）仅约7个月^[5]^。肝转移严重影响NSCLC患者的生存和预后。因此，深入探索NSCLC发生肝转移的分子机制对于探索肺癌肝转移的治疗策略具有重要意义。

SWI/SNF复合体（switch/sucrose nonfermentable chromatin-remodeling complex, SWI/SNF）是细胞内重要的染色质重塑相关的多组分复合物，主体构架由SMARCD、SMARCB1和SMARCC组成，SMARCA2/4为ATP酶，其余亚基分别组装在支架上形成多亚基复合体。复合体有三种亚型：cBAF（canonical BRM/BRG1-associated factor）、PBAF（polybromo-associated BAF）和ncBAF（non-canonical BAF），其中ARID1A/ARID1B为cBAF独有亚基^[6]^。SWI/SNF复合体利用ATP水解产生的能量介导核小体的重新定位、改变核小体结构，产生染色质重塑来改变染色质的可及性，从而调控基因表达^[7]^。肺癌及其他多个类型的肿瘤中SWI/SNF复合体基因的突变频率相对较高^[8,9]^，这些基因突变可能导致复合体功能丧失或改变，从而影响肿瘤细胞的增殖、迁移和侵袭能力并促进肿瘤转移^[10⇓⇓-13]^。在肿瘤转移过程中，转移瘤中能够检测到原发灶中不存在的其他突变，远处转移及局部复发通常与获得额外的驱动突变有关^[14]^。SWI/SNF复合体变异在肿瘤进展中具有组织环境特异性作用^[15,16]^。在不同肿瘤中，SWI/SNF对于转移复发的影响可能存在差异。例如在肝细胞癌中，ARID1A在原发性肿瘤中高表达，但在转移性病灶中ARID1A表达降低或缺失可能与迁移、侵袭和转移相关的基因转录表达有关^[16]^。而SMARCA4缺失可能与细胞恶性转化和肿瘤进展转移相关^[12]^。但SWI/SNF复合体基因突变与肺癌肝转移研究未见报道。本研究拟开展SWI/SNF复合体基因突变与肺癌肝转移之间的关系及其分子机制的研究，为探索疾病机制及治疗相关新靶点提供依据。

## 1 材料与方法

### 1.1 细胞培养

肺癌细胞系NCI-H460、NCI-H23和NCI-H1299均来自于广东省肺癌研究所细胞库，实验前进行STR鉴定（上海富衡生物），同时使用Takara支原体检测试剂盒（CY232）进行支原体检测，确保细胞系正确且无支原体污染后，使用RPMI Medium 1640 basic（1×）培养基（Gibco）和10%胎牛血清（Gibco, 1932594C）于5% CO_2_、37 ^o^C无菌细胞培养箱中培养。

### 1.2 细胞DNA提取及全外显子组测序（whole-exome sequencing, WES）

采用细胞基因组DNA提取试剂盒（Tiangen Biotech, DP304）提取细胞DNA。使用吉因加Gene+Seq-2000进行WES检测细胞基因突变状态。

### 1.3 细胞RNA提取及测序

使用RNA提取试剂盒（QIAGEN RNeasy Mini Kit, 74104 and 74106）提取细胞RNA。吉因加公司采用真核RNA测序技术应用高通量测序仪（Illumina NovaSeq6000）对3株肺癌细胞进行测序。

### 1.4 Western blot检测蛋白表达

使用BCA试剂盒（Solarbio, PC0020）检测蛋白浓度。使用One-Step PAGE Gel Fast Preparation Kit（6%）（Vazyme, E301-01）凝胶进行电泳后将蛋白转移到PVDF膜（0.45 μm, Thermo Scientific^TM^, 88518），TBST（Absin, abs952-1L）洗膜，脱脂奶粉（Solarbio, D8340）封闭90 min，4 ^o^C过夜孵育一抗：ARID1A（1:1000, CST, 12354S），ARID1B（1:1000, CST, 92964），SMARCA4（1:10000, Abcam, ab110641），SMARCA2（1:1000, CST, 11966），ARID2（1:1000, CST, 82342），PBRM1（1:1000, CST, 38439），Vinculin（1:1000, CST, 13901S），APOBEC3B（1:500, Proteintech, 14559-1-AP），ALDH1A1（1:1000, CST, 54135）。TBST洗膜，室温孵育二抗1 h：Anti-rabbit IgG（1:2000, CST, 7074），使用Clarity Max Western ECL Substrate（BIO-RAD, 1705062）显影液显影，用ImageJ软件计算分析条带灰度值。

### 1.5 病毒包装和细胞转染示踪细胞模型构建

使用293T细胞和Lipofectamine 2000（Invitrogen^TM^, 11668030）以及包装质粒体系：pWPXLd-luciferase-EGFP（中国科学院广州生物医药与健康研究院）9 μg+质粒psPAX2（addgene, 12260）12 μg+PMD 2 g（addgene, 12259）3 μg进行GL（EGFP+Luciferase）病毒包装，使用成功包装的GL病毒和1×polybrene（GLPBIO, GC19206）对NSCLC细胞系进行转染。

### 1.6 ARID1A基因稳定敲除的细胞株构建

NCI-H1299细胞系来自于广东省肺癌研究所细胞库，使用在线CRISPR设计工具（https://en.rc-crispr.com/）设计用于ARID1A敲除的sgRNA，将两个靶向位点的寡核苷酸进行退火并连接到YKO-RP006载体上（Ubigene Biosciences），使用Neon^TM^转染系统（Thermo Fisher Scientific）将含有目标sgRNA序列的YKO-RP006-hTFEB [gRNA]质粒转染到细胞中，转染24-48 h后加入嘌呤霉素进行筛选，抗性筛选后使用有限稀释法进行单克隆筛选，筛选出的ARID1A-KO克隆通过PCR、Sanger测序和Western blot进行验证。

### 1.7 动物实验

实验遵循美国国立卫生研究院（National Institutes of Health, NIH）《实验动物护理和使用指南》，经中国科学院广州生物医药与健康研究院动物护理和使用指南委员会批准。6-8周龄同性别NSI小鼠（中国科学院广州生物医药与健康研究院）随机分组进行NSCLC细胞左心室注射，每组6只，至少进行3次重复实验。使用1.25%阿佛丁（易核，M2910）经腹腔注射进行麻醉。监测小鼠活动、饮食和体重，使用D-Luciferin、Sodium Salt D-荧光素钠盐（YEASEN, 40901ES01）进行小动物活体成像检测肿瘤细胞信号强度，晚期使用球后静脉空气注射行安乐死。观察肿瘤转移情况，肝脏在4%多聚甲醛固定液中固定24 h，冲洗30 min，保存在75%酒精中。

### 1.8 苏木素-伊红（hematoxylin-eosin, HE）染色和免疫组化（immunohistochemistry, IHC）分析

标本固定后，制备石蜡切片，进行HE染色和IHC分析。提前1天进行烤片，次日脱蜡水化后用抗原修复液（GTR, GT100411）120 ^o^C修复3 min，冷却后3% H_2_O_2_孵育10 min消除内源性酶。PBS浸洗后，5% BSA（Solarbio, A8010）室温封闭1 h，随后室温孵育一抗1 h：载脂蛋白质B mRNA编辑催化亚基3B（apolipoprotein B mRNA editing enzyme catalytic subunit 3B, APOBEC3B）（Proteintech, 14559-1-AP, 1:150），醛脱氢酶家族1A1（aldehyde dehydrogenase 1 family member A1, ALDH1A1）（CST, 54135, 1:200）。PBS浸洗后，IHC二抗试剂盒（Absin, abs996）室温孵育30 min，PBS浸洗后，DAB显色试剂盒进行显色，经苏木素复染，透明后用中性树脂封片。置于光学显微镜下观察标本，计算阳性细胞数和染色强度，利用H-score进行蛋白表达水平分析。

### 1.9 统计学分析

符合正态分布的计量资料结果以均数±标准差（Mean±SD）表示，使用GraphPad Prism（V.9.0）和RStudio软件进行统计分析和可视化作图。满足正态分布且方差齐两组独立变量之间的比较采用Student 's t检验，多组独立变量比较采用One-way ANOVA单因素方差分析。若变量不服从正态分布，则采用Mann-Whitney U检验。以P<0.05定义为有统计学差异，所有检验均为双侧检验。

## 2 结果

### 2.1 肺癌细胞株SWI/SNF复合体基因突变分析及携带示踪报告基因细胞模型建立

为了解细胞中SWI/SNF复合体基因突变情况，我们对H1299、H23和H460细胞进行了WES分析，结果显示，在SWI/SNF重要的6个亚基中，H460仅存在ARID1A功能失活突变（In_Frame_Del），H23同时出现了SMARCA2功能失活突变（In_Frame_Del）、ARID2错义突变（Missense_Mutation）以及SMARCA4功能失活突变（Nonsense_Mutation）和错义突变（Missense_Mutation）；H1299细胞未检测到任何SWI/SNF复合体基因突变（表1）。Western blot分析显示，H1299细胞中SMARCA4蛋白表达水平较低，其余亚基蛋白表达正常，H23细胞中ARID1A、ARID1B和SMARCA2蛋白表达缺失，SMARCA4、ARID2和PBRM1蛋白表达水平较低，而H460细胞中仅观察到SMARCA2蛋白的表达，其余5个亚基的蛋白均未被检测到（图1A）。为顺利进行小动物活体成像，我们对细胞进行了携带GL（EGFP+Luciferase）基因的慢病毒感染，使其成功表达了EGFP和萤火虫荧光素酶，图1B展示了感染效率大于90%的细胞图像。

**表1:** H1299、H23和H460细胞中SWI/SNF复合体基因突变情况

Gene	H1299	H23	H460
ARID1A			In_Frame_Del
ARID1B			
SMARCA4	Nonsens_Mutation; Missense_Mutation		
SMARCA2	In_Frame_Del		
ARID2	Missense_Mutation		
PBRM1			

**图1:**
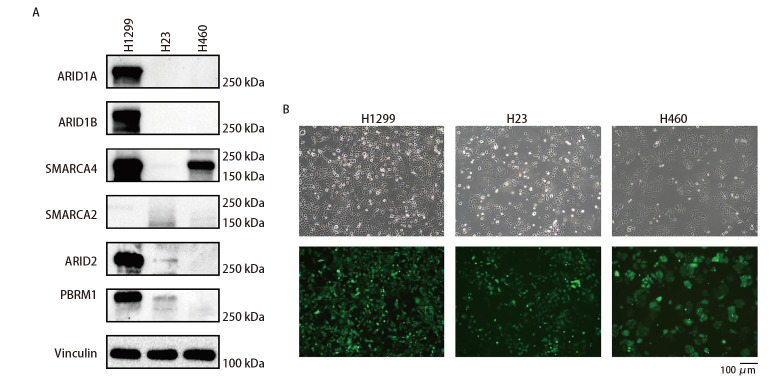
SWI/SNF复合体基因突变、蛋白表达和病毒感染的细胞特征分析。A：SWI/SNF基因蛋白表达水平；B：H1299、H23和H460细胞感染EGFP-Luciferase病毒后照片

### 2.2 SWI/SNF复合体变异细胞在NSI小鼠体内促进肝转移发生

为探索不同SWI/SNF复合体基因突变状态的肺癌细胞在免疫缺陷小鼠体内的肝转移情况，我们通过左心室注射H1299、H23和H460细胞建立了肺癌肝转移模型，生物活体发光成像监测肿瘤细胞在小鼠体内的生长和转移情况，发现SWI/SNF复合体基因野生型细胞H1299组的小鼠均未发生肝转移（0.0%），SWI/SNF复合体基因突变的细胞中H23组小鼠肝转移发生率为75.0%，而H460组所有小鼠均发生了肝转移（100.0%），图2A是代表性活体成像照片。分析肝脏中肿瘤细胞负荷发现：在H460组小鼠疾病终末期即左心室注射后第14天，ARID1A失活突变的H460与SWI/SNF复合体野生型H1299（P<0.0001）和SMARCA2/4失活突变的H23（P<0.001）相比，转移到肝脏的肿瘤细胞负荷更高，而在第28天，H23转移到肝脏的肿瘤细胞负荷也显著高于H1299（P<0.001）。且随着时间的推移，与H1299相比，H23和H460组小鼠转移到肝脏中的肿瘤细胞负荷逐渐增多（P<0.001，图2B）。

**图2:**
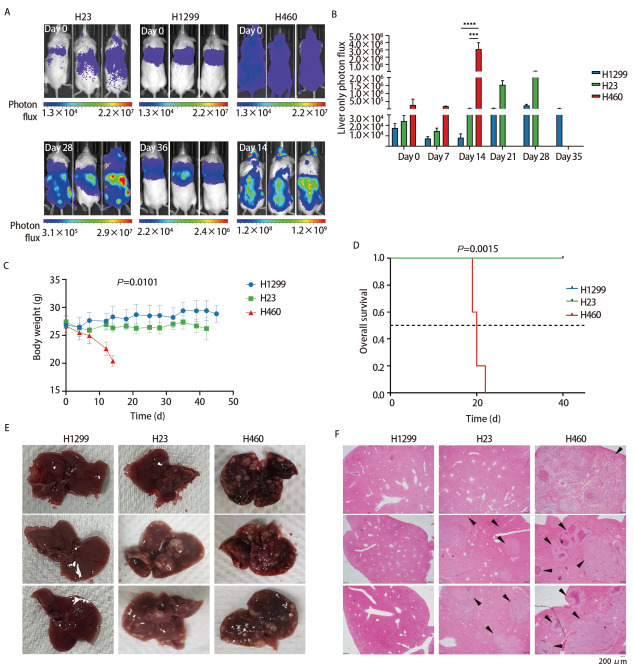
SWI/SNF复合体基因突变对免疫缺陷小鼠肺癌肝转移的影响。左心室内注射H1299、H23和H460细胞后的活体生物发光图像（A）、小鼠肝脏荧光信号值强度和变化趋势（B）、小鼠体重随时间变化趋势（C）和各组小鼠生存时间比较（D）；各组小鼠肝脏解剖学观察（E）和肝脏组织HE染色（F）。***：P<0.001；****：P<0.0001。

记录小鼠体重变化情况发现，与H1299和H23相比，H460组小鼠体重随时间变化呈明显下降趋势（P=0.0101），特别是左心室注射肿瘤细胞后的第14天，H460组小鼠体重下降趋势最为显著（图2C）。生存分析显示，与H1299和H23组小鼠相比，H460组小鼠OS显著缩短（P=0.0015），中位OS仅14天，而H1299和H23组小鼠在观察截止时间点仍存活（图2D）。

解剖学观察显示H1299组小鼠肝脏未见肿瘤病灶，H23组75%的小鼠肝脏可见转移灶，而H460组小鼠肝脏中均可观察到转移灶且数量明显多于H23组小鼠（图2E）。小鼠肝脏石蜡标本连续切片后HE染色结果显示：H1299组小鼠肝脏切片在镜下仍未观察到肿瘤转移灶，H23组小鼠中有75.0%的个体显示肝脏存在肿瘤转移灶，而H460组小鼠所有肝脏切片均观察到肿瘤转移灶且数量最多，图2F为HE染色的代表性图像。

### 2.3 ARID1A基因敲除细胞在NSI小鼠体内促进肝转移发生

利用CRISPR/Cas9（clustered regularly interspaced short palindromic repeats）基因编辑技术构建了ARID1A基因稳定敲除的细胞株H1299 sgARID1A。Western blot分析ARID1A蛋白表达情况，结果显示H1299 sgARID1A细胞中ARID1A蛋白表达缺失，而H1299正常对照（normal control, NC）组细胞中ARID1A蛋白正常表达（图3A）。随后，对两株细胞进行了携带GL基因的慢病毒感染，使其成功表达EGFP和萤火虫荧光素酶，挑选感染效率大于90%的细胞进行动物实验（图3B）。

**图3:**
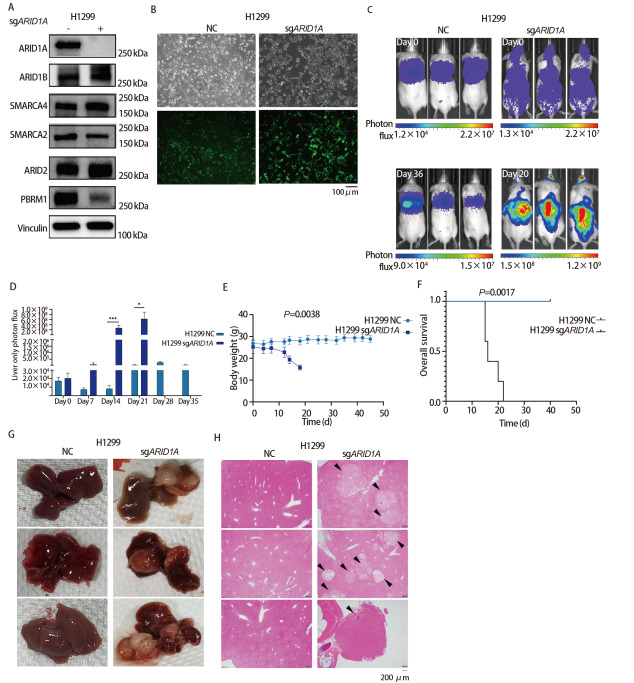
H1299细胞中敲除ARID1A基因对肺癌肝转移的影响。Western blot分析转染vector或sgARID1A的H1299中SWI/SNF基因蛋白表达水平（A）；感染EGFP-Luciferase病毒后的细胞图像（B）；左心室内注射H1299 NC和H1299 sgARID1A细胞后的活体生物发光图像（C）、小鼠肝脏荧光信号值强度和变化趋势（D）、小鼠体重随时间变化趋势（E）和各组小鼠生存时间（F）比较；各组小鼠肝脏解剖学观察（G）和肝脏组织HE染色（H）。*：P<0.05；***：P<0.001。

使用H1299 NC和H1299 sgARID1A细胞经NSI小鼠左心室注射建立肺癌肝转移模型，活体成像显示H1299 NC组小鼠均未发生肝转移，而H1299 sgARID1A组小鼠全部发生了肝转移（图3C）。肝脏肿瘤细胞负荷分析显示H1299 sgARID1A组小鼠在左心室注射后第14天（P<0.001）和第21天（P=0.010）的肝脏肿瘤细胞负荷均显著高于H1299 NC组小鼠，随着时间的推移H1299 sgARID1A组小鼠转移到肝脏中的肿瘤细胞负荷也在逐渐增多（图3D）。与H1299 NC组相比，H1299 sgARID1A组小鼠体重随时间变化呈明显下降趋势，特别是在肿瘤终末期（P=0.0038，图3E）。生存分析显示，与H1299 NC组相比，H1299 sgARID1A组小鼠OS也显著缩短（P=0.0017，图3F）。

H1299 NC组小鼠肝脏肉眼未见肿瘤病灶，H1299 sgARID1A组所有小鼠肝脏均可见转移灶（图3G）。小鼠肝脏石蜡标本连续切片后HE染色结果显示：H1299 NC组小鼠肝脏切片在镜下仍未观察到肿瘤转移灶，H1299 sgARID1A组小鼠所有个体均显示肝脏存在肿瘤转移灶（图3H）。

### 2.4 SWI/SNF复合体基因突变调节ALDH1A1和APOBEC3B的表达

为探索SWI/SNF复合体基因突变对肺癌细胞基因表达谱的影响和其可能的促进肝转移的分子机制改变，我们对细胞进行了RNA-Seq和差异基因表达分析，SWI/SNF突变细胞H23和H460相较于野生型细胞H1299有3417个基因表达上调，2554个基因表达下调，与H1299 NC相比，H1299 sgARID1A细胞有4204个基因上调，3171个基因下调，这些表达的变化呈现在火山图中（图4A和图4B），将两组差异基因取交集后得到共同上调的基因1628个和共同下调的基因1127个（图4C）。差异表达基因聚类分析显示SWI/SNF复合体基因突变和野生型细胞间的基因表达谱存在显著差异，其中ALDH1A1和APOBEC3B是比较重要的与肺癌细胞干性、肿瘤进展、侵袭和转移密切相关的基因，H460和H1299 sgARID1A中ALDH1A1显著高表达，APOBEC3B在H23和H1299 sgARID1A中表达较高，其次为H460，而在H1299中不表达（图4D）。Western blot验证结果显示，ALDH1A1蛋白仅在H460中高表达，在H1299和H23中均不表达，但APOBEC3B的表达在三者之间未观察到显著差异（图4E）。而相比于H1299 NC细胞，ALDH1A1和APOBEC3B均在H1299 sgARID1A中表达较高（图4F）。

**图4:**
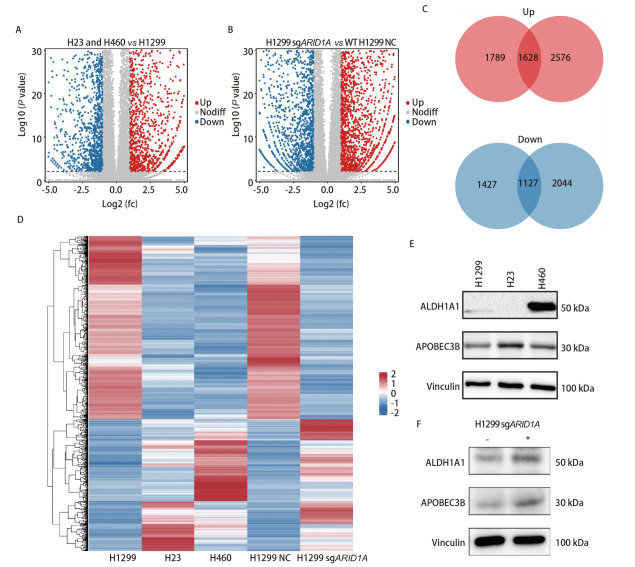
SWI/SNF复合体基因突变影响肺癌肝转移的分子机制。A：SWI/SNF复合体突变细胞H23和H460与SWI/SNF野生型细胞H1299相比的差异基因表达火山图；B：H1299 NC与H1299 sgARID1A相比的差异基因火山图；C：两组上调（上）和下调（下）的差异基因交集；D：差异基因表达热图；E，F：Western blot分析重要差异表达基因在细胞内的蛋白表达水平。

### 2.5 SWI/SNF复合体基因突变的肝转移灶中ALDH1A1和APOBEC3B蛋白表达水平分析

为进一步验证SWI/SNF复合体基因突变对相关蛋白表达的影响及其在增加肝转移发生率中的作用机制，我们通过IHC染色评估了差异基因ALDH1A1和APOBEC3B在小鼠肝转移灶肿瘤组织中的蛋白表达水平。

H1299组小鼠肝脏中无转移灶，经IHC验证，ALDH1A1和APOBEC3B蛋白分别在H23和H460组小鼠肝转移灶中的表达与RNA-Seq结果一致。ARID1A失活突变的H460组小鼠肝转移灶中ALDH1A1的蛋白表达水平显著高于SMARCA2/4失活突变的H23组（158.5 vs 0.0，P<0.0001，图5A），APOBEC3B蛋白在SWI/SNF复合体基因突变的两组细胞H460和H23组小鼠的肝转移灶中均有表达，但其表达水平在两组间未见显著差异（13.0 vs 10.0，P=0.847，图5B）。可见在SWI/SNF复合体基因突变的细胞中，ARID1A失活突变的细胞H460可能主要通过上调ALDH1A1的表达来促进肝转移的发生，而SMARCA2/4失活突变的细胞H23发生肝转移的机制可能与APOBEC3B的表达上调有关。

**图5:**
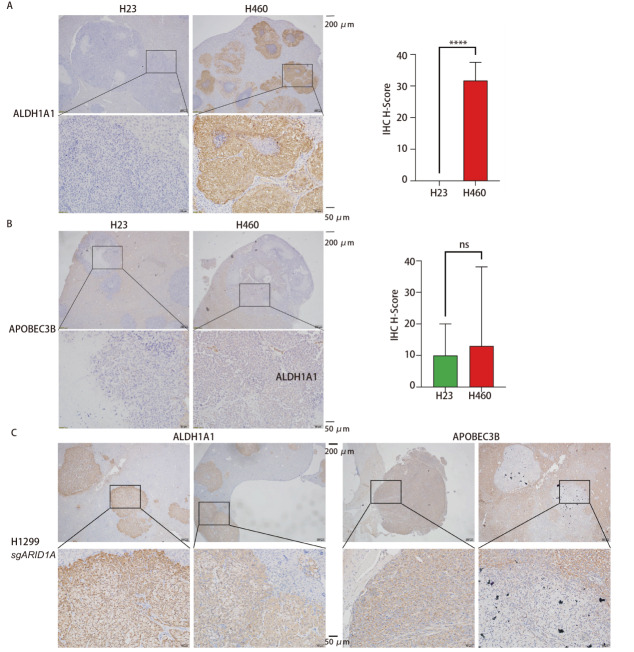
SWI/SNF复合体基因突变对肝转移灶中ALDH1A1和APOBEC3B蛋白表达水平的影响。H23和H460组小鼠肝转移灶ALDH1A1（A）和APOBEC3B（B）分子免疫组化染色代表图以及H_Score统计分析；H1299 sgARID1A组小鼠肝转移灶ALDH1A1和APOBEC3B分子免疫组化染色代表图（C）。****：P<0.0001；ns：无统计学差异。

H1299 NC组小鼠肝脏也无转移灶，经IHC染色发现，ALDH1A1蛋白在H1299 sgARID1A组小鼠肝脏转移灶中高表达，而APOBEC3B蛋白也在H1299 sgARID1A组小鼠肝转移灶中有表达（图5C），提示在H1299 sgARID1A细胞中ARID1A基因缺失也可能通过上调ALDH1A1的表达而促进小鼠肝转移的发生。

## 3 讨论

随着对SWI/SNF复合体在癌症中作用的认知加深，亟需适宜的临床前模型以探究其突变与临床结果的关联。我们鉴定了常见肺癌细胞系及其SWI/SNF复合体基因突变图谱，从基因组、转录组和蛋白水平进行分析，为细胞模型的选择和研究突变与临床观察结果关系的研究提供了参考。本研究探索了免疫缺陷小鼠中SWI/SNF复合体基因突变在肺癌肝转移中的作用和机制，全外显子组测序显示H460仅存在ARID1A失活突变，H23同时存在SMARCA2/4的失活突变和ARID2错义突变，而H1299中未检测到SWI/SNF复合体基因突变，与先前研究^[17⇓-19]^数据一致，强调不同细胞中SWI/SNF复合体基因突变的异质性，为深入探究其在肺癌发展中的作用提供线索。

动物实验证实SWI/SNF复合体基因突变促进肺癌细胞生长和转移。突变型细胞H23和H460在小鼠体内引发肝转移，而野生型细胞H1299无肝转移，其中H460组小鼠肝脏中肿瘤负荷高于H23组小鼠，突出了ARID1A失活突变影响肺癌细胞转移能力，体重和生存监测进一步证实突变细胞导致了更严重的疾病进展。而与亲本细胞H1299 NC相比，ARID1A基因敲除的细胞H1299 sgARID1A也促进了NSI小鼠肝转移的发生，同时H1299 sgARID1A组小鼠也表现出更严重的肿瘤进展。SMARCA4或ARID1A缺失与多种癌症进程有关，如病理性MYC信号传导、DNA修复缺陷和细胞周期控制受损^[20⇓⇓-23]^。尽管小鼠肺腺癌模型尚未详细研究ARID1A失活突变的效应，而SMARCA4失活突变可促进已恶性转化的细胞进一步生长，但其环境特异性效应尚未完全阐明^[24]^。ARID1A突变在小鼠肿瘤模型和肺切除患者癌前病变中均存在，但在更晚期的肿瘤疾病中，ARID1A缺失也可能促进疾病进展^[25]^。

为了进一步揭示SWI/SNF复合体基因突变对细胞表达谱的影响，本研究开展了基于RNA-Seq的基因表达谱差异分析。突变型细胞H23和H460相对于野生型细胞H1299以及ARID1A基因敲除的细胞H1299 sgARID1A相对于亲本细胞H1299 NC差异表达的基因调控了多个与肿瘤进展、侵袭和转移密切相关的基因的表达，包括ALDH1A1和APOBEC3B。蛋白印迹和IHC染色结果进一步验证了差异基因的蛋白产物表达。这些发现为深入探究SWI/SNF复合体基因突变对肺癌细胞行为的影响提供了关键线索。

ALDH1A1参与细胞代谢和氧化应激反应，与肿瘤干细胞特性、细胞迁移和侵袭及预后不良有关^[26⇓⇓-29]^。我们发现SWI/SNF复合体基因ARID1A失活突变与ALDH1A1高表达显著关联，特别是H460细胞和ARID1A基因敲除的细胞H1299 sgARID1A引发的肝转移灶中ALDH1A1表达显著上调，可能是肿瘤细胞对外界环境变化的适应策略之一，提示ARID1A突变可调节ALDH1A1的表达，影响肺癌细胞的转移能力，这一发现为深入研究SWI/SNF复合体基因ARID1A与ALDH1A1的相互作用及其在肺癌转移中的精细调控提供了新方向。既往研究^[30⇓⇓⇓-34]^已经提到了ALDH1A1在肿瘤转移中的作用，ALDH1A1高表达与肿瘤患者的分期和远处转移密切相关，尤其是在乳腺癌和消化道肿瘤中。研究^[35]^表明S100A9可在奥希替尼耐药的肺癌细胞中上调ALDH1A1的表达并激活维甲酸（retinoic acid, RA）信号通路，而高表达胞内S100A9的肺癌细胞可逃逸奥希替尼并引发脑转移的复发。同样，ALDH1A1还可通过增强BRCA2卵巢癌细胞的DNA修复，促进PARP抑制剂的耐药性^[36]^。然而，对于ALDH1A1与SWI/SNF复合体基因突变之间的关联，目前的研究仍有限。本研究初步证实了这一关联，强调ALDH1A1在SWI/SNF复合体基因突变肺癌细胞转移中的潜在作用。APOBEC3B是APOBEC家族中的重要成员，该家族成员的酶活性对适应性免疫和先天性免疫反应都至关重要，而APOBEC3B的表达在多种癌症尤其是NSCLC中上调，且基因组体细胞突变也更频繁^[37]^，APOBEC相关突变在NSCLC发展的后期更多见，且与肿瘤进展和转移相关^[38,39]^。我们的研究结果初步发现APOBEC3B在SWI/SNF复合体基因尤其是ARID1A基因失活突变的细胞和肝转移灶中表达上调，提示了SWI/SNF复合体基因的突变可能也通过上调APOBEC3B的表达从而促进肺癌细胞的转移。

本研究揭示了SWI/SNF复合体基因突变能够促进肺癌细胞肝转移的发生。我们还发现了与肿瘤进展密切相关的基因，SWI/SNF复合体基因突变可能经由上调ALDH1A1和APOBEC3B的表达而发挥促进转移的作用。下一步拟详细研究SWI/SNF复合体基因功能变异与ALDH1A1/APOBEC3B表达的调控机制，为肺癌肝转移的机制阐述和潜在治疗靶点探索提供科学数据。

**Competing interests**

The authors declare that they have no competing interests.
